# Decreased Caffeine-Induced Locomotor Activity via Microinjection of CART Peptide into the Nucleus Accumbens Is Linked to Inhibition of the pCaMKIIa-D_3_R Interaction

**DOI:** 10.1371/journal.pone.0159104

**Published:** 2016-07-12

**Authors:** Qiang Fu, Xiaoyan Zhou, Yun Dong, Yonghong Huang, Jianhua Yang, Ki-Wan Oh, Zhenzhen Hu

**Affiliations:** 1 Department of Respiration, The Fourth Affiliated Hospital, Nanchang University, Nanchang, Jiangxi, China; 2 Department of Respiration, Department Two, Jiangxi Provincial People’s Hospital, Nanchang, Jiangxi, China; 3 Department of Pathophysiology, College of Medicine, Nanchang University, Nanchang, Jiangxi, China; 4 Department of Breast Surgery, Jiangxi Tumor Hospital, Nanchang, Jiangxi, China; 5 Department of Physiology, College of Medicine, Nanchang University, Nanchang, Jiangxi, China; 6 College of Pharmacy, Chungbuk National University, Cheongju, Republic of Korea; Nanjing Agricultural University, CHINA

## Abstract

The purpose of this study was to characterize the inhibitory modulation of cocaine- and amphetamine-regulated transcript (CART) peptides, particularly with respect to the function of the D_3_ dopamine receptor (D_3_R), which is activated by its interaction with phosphorylated CaMKIIα (pCaMKIIα) in the nucleus accumbens (NAc). After repeated oral administration of caffeine (30 mg/kg) for five days, microinjection of CART peptide (0.08 μM/0.5 μl/hemisphere) into the NAc affected locomotor behavior. The pCaMKIIα-D_3_R interaction, D_3_R phosphorylation and cAMP/PKA/phosphorylated CREB (pCREB) signaling pathway activity were measured in NAc tissues, and Ca^2+^ influx and pCaMKIIα levels were measured in cultured NAc neurons. We found that CART attenuated the caffeine-mediated enhancement of depolarization-induced Ca^2+^ influx and CaMKIIα phosphorylation in cultured NAc neurons. Repeated microinjection of CART peptides into the NAc decreased the caffeine-induced enhancement of Ca^2+^ channels activity, pCaMKIIα levels, the pCaMKIIα-D_3_R interaction, D_3_R phosphorylation, cAMP levels, PKA activity and pCREB levels in the NAc. Furthermore, behavioral sensitization was observed in rats that received five-day administration of caffeine following microinjection of saline but not in rats that were treated with caffeine following microinjection of CART peptide. These results suggest that caffeine-induced CREB phosphorylation in the NAc was ameliorated by CART peptide due to its inhibition of D_3_R phosphorylation. These effects of CART peptides may play a compensatory role by inhibiting locomotor behavior in rats.

## Introduction

Caffeine increases alertness and enhances the locomotor performance of humans and produces hyperactivity in other animal species [1]. These sensitization effects are triggered by an increase in the levels of intracellular Ca^2+^/calmodulin-dependent kinase (CaMK) signaling and by phosphorylation of cyclic adenosine 5’-monophosphate (cAMP) response element-binding protein (CREB) [2–5], which depends on the adenosine-mediated modulation of the dopaminergic (DA) system [6,7]. Previously, it was shown that intra-accumbal injection of an inhibitor of Ca^2+^ channels or CaMKIIα attenuated the effects of psychostimulants on behavioral sensitization [8–11]. Increasing CREB phosphorylation in the rat NAc using a PKA activator enhanced the rewarding effects of cocaine, whereas decreasing CREB phosphorylation using a PKA inhibitor reduced cocaine self-administration [5]. Several reports in rodents have demonstrated the ability of D_3_R-selective partial agonists and antagonists to attenuate the discriminative stimulatory effects of cocaine [12]. Moreover, CaMKIIα directly interacts with a selective serine residue of D_3_R in a Ca^2+^- and autophosphorylation-sensitive manner. The interaction of CaMKIIα with D_3_Rs in accumbal neurons *in vivo* has a significant role in regulating behavioral responsiveness to cocaine [13].

Cocaine- and amphetamine-regulated transcript (CART) peptides are neuropeptides that are highly expressed within the NAc, the hypothalamus and the ventral tegmental area (VTA), and these peptides mediate drug reward and reinforcement [14–16]. Treatment of rats and mice with cocaine or amphetamine results in the production of alternatively spliced variants and post-translational cleavage of CART, leading to the production of several bioactive fragments, such as CART 55–102 and CART 62–102 [17]. Increased CART expression in the NAc may be partially mediated by D_3_ receptor (D_3_r) activity [18]. CREB binding sites in the CART promoter sequence are involved in the expression of the CART gene [19]. Mutations at the CRE binding site of the CART promoter decrease promoter activity [20]. Moreover, CART expression appears to be regulated by the dopamine receptor-Ca^2+^/cAMP/protein kinase A (PKA)/phosphorylated CREB (pCREB) signaling pathway in multiple cell lines [21,22]. *In vivo* studies have demonstrated that a D_1_ or D_2_/D_3_ receptor antagonist can block the over-expression of CART induced by ethanol in the rat NAc [23]. Intra-accumbal activation of cAMP/PKA/pCREB signaling has been shown to stimulate the phosphorylation of CREB, resulting in an increase in the levels of CART mRNA and peptide in the rat NAc [24]. A few studies have also indicated that CART peptide in the NAc attenuates the locomotor effects of psychostimulants [25–27]. Thus, dopamine receptors and CREB can control the expression of CART peptides to some extent. In turn, CART peptides can regulate locomotor activity via dopamine receptor and cAMP/PKA/pCREB signaling pathway activities. Our previous report showed that cocaine- and caffeine-induced sensitization was expressed on the 5^th^ day of administration and that CREB phosphorylation and CART peptide expression peaked on the 5^th^ day [28,29]. In the present study, we examined the effects of CART peptides on locomotor activity and CREB phosphorylation on the 5^th^ day of caffeine administration.

## Materials and Methods

### Animals and chemicals

Adult male Sprague-Dawley (SD) rats weighing between 260 g and 280 g were used. The animals were housed one per cage in the animal care facility and provided with water and food available *ad libitum* under an artificial 12/12 (h) light/dark cycle (lights on at 07:00) and constant temperature (22±2°C). All experiments animals were maintained in accordance with the National Institutes of Health Guide for the Care and Use of Laboratory Animals (NIH publication No. 8023, revised 1978) (S1 Fig). The protocol was approved by the Committee on the Ethics of Animal Experiments of the University of Nanchang (Permit Number: 2010–0002). All surgeries were performed under sodium pentobarbital anesthesia, and all efforts were made to minimize animal suffering. No animals became severely ill or died at any time prior to the experimental endpoint. Caffeine was purchased from Sigma Chemical Company (St. Louis, MO). CART 55–102 peptide was purchased from American Peptide Company (cat #1305195T, Vista, CA). All chemicals were dissolved immediately before use in physiological saline.

### Experimental procedures

All experiments were conducted in a randomized, balanced repeated-measures design such that each rat received all the treatments during the experiments. Adult male SD rats were randomly divided into 4 groups of 8 rats each. A separate group of rats was used for each behavioral experiment. Each experiment was repeated at least three times. A total of 192 rats received pretreatment with one bilateral accumbal infusion (saline or 0.08 μM CART 55-102/hemisphere). After the administration of chemicals, the locomotor activity of the 96 rats was immediately measured using an infrared photocell-based automated Opto-Varimex-Micro apparatus. The locomotor activity of the other 96 rats was measured using a tilting-type ambulometer. Prior to the administration of chemicals, the rats were placed in the experimental chamber for a 30-min habituation period. Behavioral sensitization was induced in each rat immediately after oral administration of 30 mg/kg caffeine for five consecutive days according to our previous methods [30–32] (S2 Fig). Then, adult male SD rats were randomly divided into 4 groups of 4 rats each. Each experiment was repeated at least three times. After 30 min of treatment followed by decapitation, a total of 48 NAc tissues were dissected in ice-cold saline. Ca^2+^ channel expression, CaMKIIα phosphorylation, the phosphorylated CaMKIIα (pCaMKIIα)-D3R interaction, D3R phosphorylation and CREB phosphorylation were measured using western blot analysis. The cAMP levels were measured using enzyme-linked immunosorbent assay (ELISA). PKA activity was measured using radiometric analysis. Post-natal NAc tissues were cultured for the measurement of Ca^2+^ influx using the Fluo-4 NW Ca^2+^ assay. A separate group of cells was used for each experiment. The cells were divided into two groups of 4 samples each. The cells were treated (with saline or 10 mM caffeine) for at least 30 min, followed by application of 30 mM KCl. The cells were divided into four groups of 4 samples each. The cells were pretreated (with saline or 1 μM CART 55–102) for 5 min and then treated (with saline or 10 mM caffeine) for at least 30 min, followed by application of 30 mM KCl.

### Surgical and infusion procedures

At least 1 week after arrival, the rats were anesthetized with pentobarbital sodium (42 mg/kg, i.p.; Sigma Co., St. Louis, MO). A bilateral stainless steel guided cannula assembly (22-gauge; Plastics One, Roanoke, VA) was implanted above the NAc using a motorized stereotaxic StereoDrive system (Neurostar Co., Sindelfingen, Germany). The target coordinates relative to Bregma were A/P + 1.7, L/M ± 1.6, and D/V -7.5 [33]. Guide cannulas were anchored in place using dental acrylic and two stainless steel screws, which were inserted into the skull. Amoxicillin powder (0.25 g/capsule, Baiyun Mountain Pharmaceutical Co., Ltd., Guangzhou, China) was placed on the skull to protect against intracranial infection. Dummy cannulas that extended 0.5 mm beyond the tip of the cannulas were inserted to prevent blockage, and a dust cap was attached to the top of the cannula assembly. The rats were allowed to recover from surgery for at least two weeks before the start of the experiment.

Stainless steel injector cannulas (28-gauge; Plastics One) that projected 2 mm beyond the tip of the guide cannulas were used for infusions. These cannulas were connected to 10-μl syringes (Neurostar Co., Sindelfingen, Germany) via polyethylene-10 tubing. A microinjection system (Neurostar Co., Sindelfingen, Germany) was used for fluid delivery. For each infusion, rats were confined to a small polyethylene box. Bilateral infusions into the NAc were administered as a 0.5-μl volume per side over a 30-s duration. After the infusions, the injector cannula was removed, the dummy cannula was retracted, and the dust cap was secured.

### Measurement of locomotor activity

The locomotor behavior of the rats was in an open field under illuminated conditions. The rats were placed in an activity chamber and were habituated to the chamber environment for 30 min before each experiment. Three sensor pairs that were positioned in the X, Y (horizontal) and Z (vertical) dimensions were assigned to each cage. Infrared beam interruptions caused by the presence of the rats were transferred from all sensors to a computer equipped with operating software. In addition, the locomotor activities of the rats were measured using a tilting-type ambulometer. Each rat was placed in the activity cage (20 cm in diameter, 18 cm in height). Chemicals were administered after an adaptation period of 10 min. Each rat was first allowed to perambulate for 10 min in the activity cages, followed by a 1-h test period immediately after caffeine administration. The development of sensitization over 5 days was evidenced by an increase in the behavioral sensitization response to caffeine relative to the response on the 1^st^ day of caffeine administration.

### Histology

After completion of the experiments, each rat was anesthetized and decapitated. After each brain was removed, it was immediately frozen for slicing on a cryostat. Each brain was sliced into 50-μm-thick coronal sections through the area of the guided cannula. These sections were mounted onto slides, stained with toluidine blue, and examined under a microscope to localize the tip of the injector cannula. Ten animals were removed from the study due to misplacement of the cannulas.

### Western blot analysis

NAc cells or tissues were dissected in ice-cold saline and homogenized in Protein Extraction Solution. The supernatants were collected and stored at -20°C. The protein concentration of the supernatant was determined via the Bradford method, using bovine serum albumin as the standard. Equal amounts of proteins were separated on an SDS/10% polyacrylamide gel and then transferred to a polyvinylidene difluoride (PVDF) membrane. The membrane was blocked with 0.5% non-fat milk in TBS-T [10 mM Tris (pH 8.0) containing 0.05% Tween-20], followed by three washes in TBS-T. The membranes were incubated with specific antibodies using the SNAP i.d. system (Millipore Co. Bedford, MA). Anti-pCaMKIIα (1:67 dilution, Santa Cruz Biotechnology, Santa Cruz, CA), anti-pCREB (1:167 dilution, Abcam, Cambridge, MA), anti-pan-PMCA (ATP2B) (Cat #9R208306-1, 1:83 dilution, Abcam, Cambridge, MA) and anti-GAPDH antibodies (1:333 dilution, Santa Cruz Biotechnology, Santa Cruz, CA) were used in this study. The membrane was then incubated with the corresponding horseradish peroxidase-conjugated immunoglobulin G antibody. Immunoreactivity was detected by incubating the membrane in ECL-Plus chemiluminescent substrate. Chemiluminescence was observed using the FUSION-FX7 imaging system (Vilber Lourmat Co., Cedex, France). Band intensity (OD) was quantified via densitometry using FUSION-CAPI analysis software.

### In vitro binding assay

The pCaMKIIα-D_3_R interaction was measured according to the modified methods of Liu et al. [13]. The D_3_R protein was purified from total protein samples (500 μg) via immunoprecipitation using Protein A/G Agarose Beads (CMCTAG Inc., WI, USA) and an anti-D_3_R antibody (5 μL) (Cat #C1813 and Cat #F1307; Santa Cruz Biotechnology, Santa Cruz, CA). The concentration of D_3_R in the immunoprecipitate was determined via the Bradford method using bovine serum albumin as the standard. Equal amounts of the D_3_R immunoprecipitate were collected for western blot analysis using anti-pCaMKIIα (Cat #A2512 and Cat #E2013) and phosphoserine antibodies (Cat #B0311; 1:67 dilution, Santa Cruz Biotechnology). In a reverse coimmunoprecipitation assay, the pCaMKIIα immunoprecipitate was used for western blot analysis with the anti-D_3_R antibody (1:67 dilution).

### Analysis of cAMP levels

All procedures were performed in accordance with the conditions of our western blotting experiments. The cAMP levels were determined using a competition enzyme-linked immunoassay (ELISA) kit (Millipore Co. Bedford, MA). The absorbance was measured using a spectrofluorimeter (BMG Co., Ortenberg, Germany) with the emission set at 405 nm. Measuring absorbance with respect to the cAMP standard allows for the calculation of the absolute amount of cAMP in a sample of interest. We used 5 mg of tissue, and the results are expressed as nM/cAMP/mg tissue.

### Radiometric analysis

All procedures were performed in accordance with the conditions of our western blotting experiments. PKA was purified from total protein samples (300 μg) using the same anti-PKA antibody used in the immunoprecipitation experiments. The PKA immunoprecipitate was dissolved in assay dilution buffer for radiometric analysis. Equal amounts of the PKA immunoprecipitate were added to incubation tubes containing 1.67 μM cAMP, 0.08 mM Kemptide, 0.33 μM PKC/CaMK inhibitor cocktail, and 1.67 μCi of [γ-^32P^]ATP/magnesium/ATP cocktail to a total volume of 60 μl. The solution was incubated with shaking at 30°C for 10 min. A 25-μl aliquot was blotted onto a numbered phosphocellulose paper (p81) square, which was then washed 3 times with 0.75% phosphoric acid and once with acetone. The results were read in a Wallac 1450 MicroBeta Trilux liquid scintillation counter (Cardinal Health Co., Dublin, OH) and calculated by quantifying the counts per minute (CPM) in samples containing the PKA enzyme relative to the CPM of the control samples containing no enzyme. The results are expressed as pmol phosphate incorporated into Kemptide/min/μg protein.

### Post-natal NAc cell cultures

Post-natal NAc cells were prepared according to the modified methods of Shi and Rayport [34]. Post-natal (P1) rats were anesthetized via hypothermia on ice, and their brains were removed and placed in ice-cold phosphate-buffered saline (PBS). The forebrain was split sagittally at the midline. With the medial surface facing up, a 16 G sharp-edged cannula was used to punch out a cylinder of tissue containing the NAc using the anterior commissure to define its peripheral border (caudal/dorsal). The lateral one-third of the cylinder (cortex) and the medial one-fourth (using the lateral ventricle as a reference point) of the cylinder were removed using a scalpel blade. The middle portion was transferred to ice-cold HBSS solution (Ca^2+^- and magnesium-free). The tissue was rinsed with cold HBSS and dissociated using 0.25% trypsin-EDTA (Sigma) for 15–20 min at 37°C, followed by trituration with 22 G and 25 G needles. The cells were suspended in 10 mL of 20% fetal bovine serum (FBS) in Neurobasal growth media (Gibco, Grand Island, NY; 0.5 mM glutamine, 25 μM glutamate, 100 U/ml penicillin; 100 U/ml streptomycin) in a 50-mL conical tube and then centrifuged for 5 min (100 g). Then, B27 supplement (Gibco, Grand Island, NY) was added, and the NA cells were plated at a density of 40 000 cells/ml (100 cells/mm^2^) on poly-D-lysine-coated 96-well culture microplates in Neurobasal growth media supplemented with B27. One-half of the media was replaced with Neurobasal growth media every 3 days. All experiments were performed using cells cultured for two weeks.

### Ca^2+^ influx measurement

After completely removing the growth media from the cell cultures, the cells were incubated in Fluo-4 NW at 37°C for 30 min and then at room temperature for an additional 30 min, as described in the protocol of the Fluo-4 NW Ca^2+^ Assay Kit (Molecular Probes, Invitrogen, Eugene, OR). Repetitive fluorescence measurements were immediately recorded using a spectrofluorometer (BMG Labtech, Ortenberg, Germany) with excitation at 494 nm and emission at 516 nm. The data are represented as the relative fluorescence, F_0_/F, where F_0_ is the original fluorescence preceding Ca^2+^ application and F is the fluorescence as a function of time. The data were digitized at 6-sec intervals.

### Immunofluorescence analysis

The cells were incubated with the anti-pCaMKIIα antibody (1:50 dilution, Santa Cruz Biotechnology, Santa Cruz, CA) diluted in TBS overnight at 4°C after blocking the cells in diluted normal serum for 30 min. After three washes with TBS-T, the sections were incubated with Alexa 488-conjugated anti-rabbit IgG (1:1000 dilution, Molecular Probes, Eugene, OR) for 40 min at room temperature. Next, the cells were incubated with 4’,6-diamidino-2-phenylindole (DAPI) for 15 min at 37°C. Finally, the cells were rinsed, mounted on slides and cover-slipped for fluorescence microscopy and photography using an ApoTome microscope (Carl Zeiss, Thornwood, NY). The density of pCaMKIIα-positive cells was expressed as the number of cells per high-power field using Fusion CAPI analysis software.

### Data analysis

The results are presented as the means ± SEM. The interaction effects of CART 55–102 peptide and with caffeine and saline in cells and in rats were assessed using two-way analysis of variance (ANOVA) followed by Bonferroni’s *post hoc* test. The significance of the effects was assessed within groups using two-way ANOVA, as appropriate, followed by Dunnett’s *post hoc* test. Statistical significance was set at p<0.05.

## Results

### CART peptides decrease the locomotor activity of rats in response to caffeine

**Fig 1:**
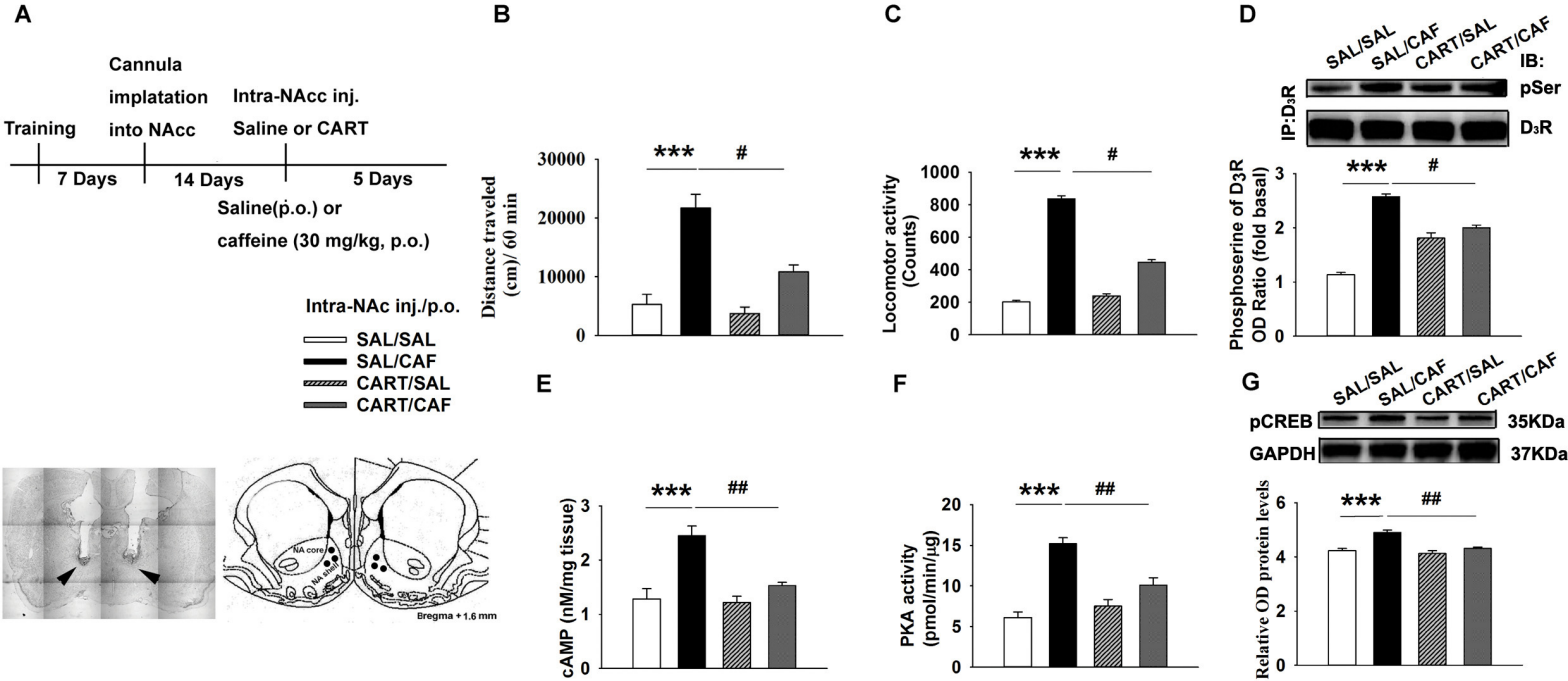
CART Peptide Inhibits Caffeine-induced Locomotor Behavior and Activation of D3R/cAMP/PKA/pCREB Signaling. Different groups were microinjected with either saline or 2 μM CART 55–102, followed by oral administration of either saline or caffeine. (**a)** Experimental timeline and location of the injection cannula tips for the rats that were included in the data analyses. The line drawings are from the work by Paxinos and Watson [33]. The numbers on the right indicate the distance in millimeters from Bregma. (**b**) and (**c**) Time-course data are shown as the group mean (+SEM) locomotor activity distance (b) and number of count (c) obtained during a 1-h test period following treatment over 5 days. (**d**) Immunoblot analysis of phosphoserine levels was performed on D3R precipitates. The membranes were scanned, and the band intensities were quantified by measuring the arbitrary OD of the phosphoserine or D3R bands. (**e**) The level of cAMP in the NAc of rats was measured using a competition ELISA. (**f**) After immunoprecipitation for the PKA protein, PKA activity was measured using a radiometric assay. (**g**) A representative western blot labeled with antibodies against pCREB. The membranes were scanned, and the band intensities were quantified by measuring the relative density (relative OD) of the immunoreactive signal of pCREB to GAPDH. The data are presented as the means ± SEM of each group (n = 4-8/group). ***p<0.001, compared to the saline group; ^+^*p*<0.05, compared to the first day of caffeine treatment; ^#^*p*<0.05 and ^##^*p*<0.01, compared to the caffeine group. The symbols indicate significant differences as revealed by two-way ANOVA followed by Bonferroni’s *post hoc* test or Dunnett’s *post hoc* test. Symbols represent different groups: □, saline (intra-NAc)-saline (p.o.); ■, saline-caffeine; ▩, CART 55–102 (0.08 μM/side)-saline; ▨, CART 55-102-caffeine.

No animals became severely ill or died at any time prior to the experimental endpoint. All injections were confirmed to be in the accumbal area at or near the desired shell. 10 animals were excluded due to an improper injection location. The locations of all 230 cannulas applied in this study are graphed in Fig 1. Compared to the control treatment, repeated oral administration of caffeine over a 5-day period following repeated microinjection of saline over a 5-day period significantly increased locomotor activity (open field tests, *n = 6–8*, *p*<0.001, Fig 1B). Moreover, the caffeine-treated rats exhibited more locomotor activity at day 5 than at day 1 (*n = 6–8*, *p*<0.05, Fig 1B). This result indicates that the rats were behaviorally sensitized to caffeine at day 5. However, this effect was partially blocked by microinjection of CART 55–102 peptide into the NAc. Following caffeine administration, locomotor activity was significantly decreased by microinjection of CART 55–102 peptide (0.08 μM/side), but not saline, into the NAc (*n = 6–8*, *p*<0.05, Fig 1B).

In experiments using a tilting-type ambulometer, repeated administration of caffeine following microinjection of saline also increased locomotor activity ([F_(3, 12)_ = 158.3, *p*<0.001, Fig 1C) and resulted in behavioral sensitization (*n = 6–8*, *p*<0.05, Fig 1C) at day 5 compared to the appropriate control. Microinjection of CART 55–102 (0.08 μM/side) into the NAc following administration of caffeine decreased locomotor activity compared with microinjection of saline following administration of caffeine (*n = 6–8*, *p*<0.05, Fig 1C). These results indicated that caffeine-induced behavioral sensitization was blocked by CART 55–102 peptide at a dose of 0.08 μM/side.

### CART peptides decrease the caffeine-mediated activation of D_3_R phosphorylation and cAMP/PKA/pCREB signaling in the NAc of rats

A series of coimmunoprecipitation and western blot analyses were conducted to measure D_3_R phosphorylation in the NAc. Caffeine increased serine phosphorylation in basal D_3_R precipitates, as detected by an antibody selective for phosphoserine (*n* = 4, *p*<0.001, Fig 1D). This increase was blocked by microinjection of CART 55–102 (0.08 μM/side) into the NAc at day 5 of caffeine administration (*n* = 4, *p*<0.05, Fig 1D). This observation indicated that the CART peptide promotes D_3_R dephosphorylation.

Based on ELISA and radiometry, repeated oral administration of 30 mg/kg caffeine followed by microinjection of saline led to increased levels of cAMP (*n* = 4, *p*<0.001, Fig 1E) and PKA activity (*n* = 4, *p*<0.001, Fig 1F) in the NAc at day 5 compared to the control treatment. However, these effects were blocked by microinjection of CART 55–102 (0.08μM/side) into this site prior to oral administration of caffeine (Fig 1E and 1F).

As demonstrated by western blot analysis, repeated administration of caffeine followed by microinjection of saline led to increased levels of CREB phosphorylation in the NAc by approximately 1.3-fold relative to those obtained via saline treatment. Nevertheless, administration of active CART 55–102 (0.08 μM/side) completely blocked the caffeine-mediated increase in CREB phosphorylation (*n* = 4, *p*<0.05, Fig 1G) at this site.

### CART peptides decrease the caffeine-induced enhancement of the Ca^2+^-evoked pCaMKIIα-D_3_R interaction in the rat NAc

**Fig 2:**
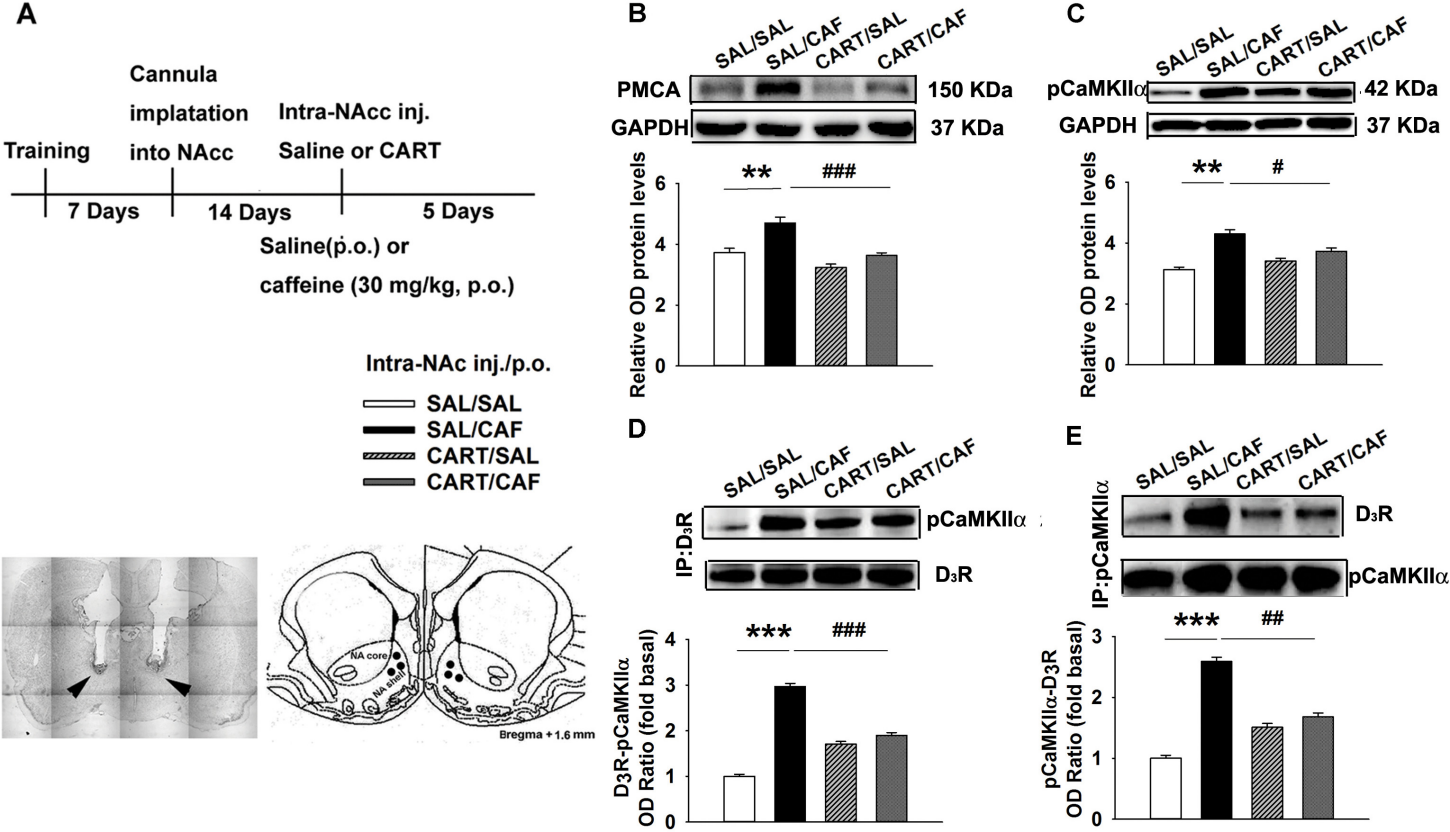
CART Peptide Decreases the Caffeine-induced Increase in the pCaMKIIα–D_3_R Interaction Level. (**a**) Experimental timeline and location of the injection cannula tips for the rats included in the data analyses. (**b**) A representative western blot labeled with antibodies against pan-PMCA. (**c**) A representative western blot labeled with antibodies against pCaMKIIα. The membranes were scanned, and the band intensities were quantified by measuring the relative density (relative OD) of the immunoreactive signals of PMCA and pCaMKIIα to GAPDH. (**d**) Immunoblot analysis of D_3_R expression was performed on pCaMKIIα immunoprecipitates. **e** Immunoblot analysis of pCaMKIIα expression was performed on D_3_R immunoprecipitates. The membranes were scanned, and the band intensities were quantified by measuring the arbitrary OD of the pCaMKIIα or D_3_R bands. The data are presented as the means ± SEM of each group (n = 4/group). ***p<0.001, compared to the saline group; ^##^*p*<0.01 and ^###^*p*<0.001, compared to the caffeine group. Symbols indicate significant differences as revealed by two-way ANOVA followed by Bonferroni’s *post hoc* test. Symbols represent different groups: □, saline (intra-NAc)-saline (p.o.); ■, saline-caffeine; ▩, CART 55–102 (0.08 μM/side)-saline; ▨, CART 55-102-caffeine.

In the western blot experiments, repeated administration of caffeine and subsequent microinjection of saline led to an increased intracellular Ca^2+^ concentration (*n* = 4, *p*<0.01, Fig 2B) and pCaMKIIα level (*n* = 4, *p*<0.01, Fig 2C) in the NAc compared with saline treatment. In contrast, the active CART peptide decreased these effects of caffeine on Ca^2+^/pCaMKIIα signaling (*n* = 4, *p*<0.001 and *p*<0.05, Fig 2B and 2C) at this site.

A series of coimmunoprecipitation and western blot analyses were conducted to measure the pCaMKIIα-D_3_R interaction in the rat NAc. A band corresponding to pCaMKIIα was clearly observed following western blot of the D_3_R immunoprecipitates (Fig 2D). In a reverse coimmunoprecipitation assay, a D_3_R-specific band was observed in the pCaMKIIα immunoprecipitates (Fig 2E). Caffeine induced an increase in the amount of pCaMKIIα in the D_3_R immunoprecipitates (*n* = 4, *p*<0.001, Fig 2D and 2E), which was in agreement with the increased amount of D_3_R in the pCaMKIIα immunoprecipitates (*n* = 4, *p*<0.001, Fig 2D and 2E). The CART 55–102 peptide blocked the caffeine-potentiated precipitation of D_3_R with pCaMKIIα (*n* = 4, *p*<0.001, Fig 2D) and of pCaMKIIα with D_3_R (*n* = 4, *p*<0.01, Fig 2E).

### CART peptides inhibit the caffeine-induced increases in Ca^2+^ influx and pCaMKIIα expression in cultured NAc neurons

**Fig 3:**
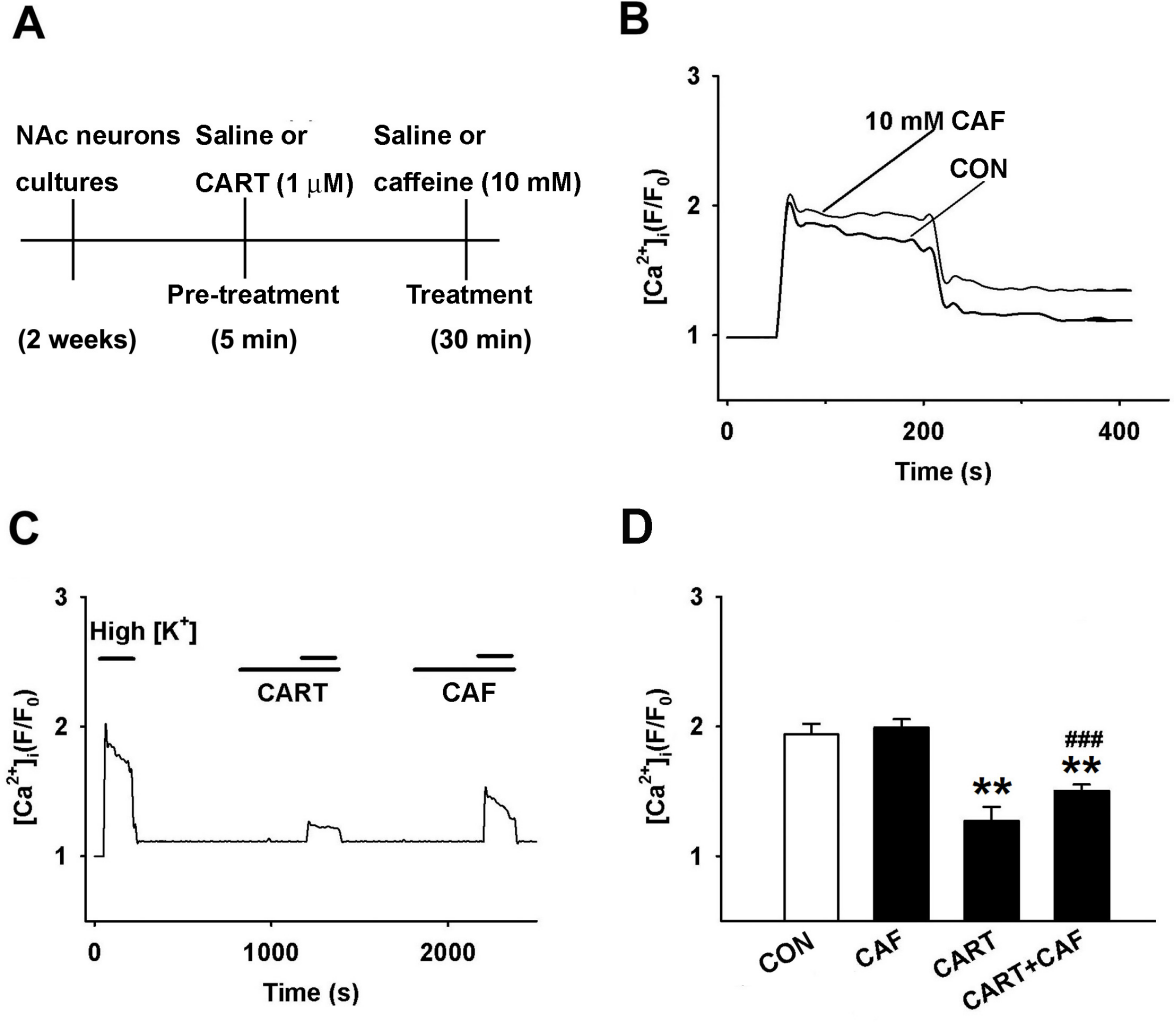
CART Peptide Decreases Caffeine-enhanced Depolarization-induced Ca^2+^ Influx in Cultured NAc Neurons. (**a**) Experimental timeline. (**b**) K^+^ depolarization-induced Ca^2+^ influxes in NAc neurons were recorded in the presence of 0 or 10 mM caffeine. (**c**) Depolarization-induced Ca^2+^ influx was recorded under control conditions after a single 5-min application of 1 μM CART 55–102 peptide and after a single 5-min application of 10 mM caffeine. High K^+^ (35 mM) application is indicated by the *short bars*, and CART 55–102 peptide application is indicated by the *long bars*. (**d**) Bar graph showing the quantification of Ca^2+^ influx in cultured NAc cells (c). The data are shown as the group means ± SEM (n = 5/group). **p<0.01 compared to the control group (one-way ANOVA followed Dunnett’s *post hoc* test). ^###^*p*<0.001, compared to the caffeine group (one-way ANOVA followed Bonferroni’s *post hoc* test).

Increases in the intracellular levels of Ca^2+^ result in the autophosphorylation of the regulatory domain of adjacent CaMKIIα subunits and, therefore, the phosphorylation of D_3_R [13]. We have previously reported that CART 55–102 peptide dose-dependently reduced the amplitude of Ca^2+^ influx that was elicited by K^+^ depolarization in Fluo-4 NW-loaded rat NAc cultured neurons [32]. In this study, we found that 10 mM caffeine potentiated the intracellular Ca^2+^ signals produced via K^+^ depolarization in cultured NAc neurons (*n* = 5, Fig 3B). However, these effects were attenuated by CART 55–102 peptide (1 μM) following application of caffeine. CART 55–102 peptide reduced the intracellular Ca^2+^ signals in NAc neurons by approximately 0.79-fold relative to that obtained after application of 10 mM caffeine followed by saline treatment. Moreover, application of 10 mM caffeine partially reversed the inhibition of Ca^2+^ transients observed in the presence of CART 55–102 peptide (*n* = 5, Fig 3C and 3D).

**Fig 4:**
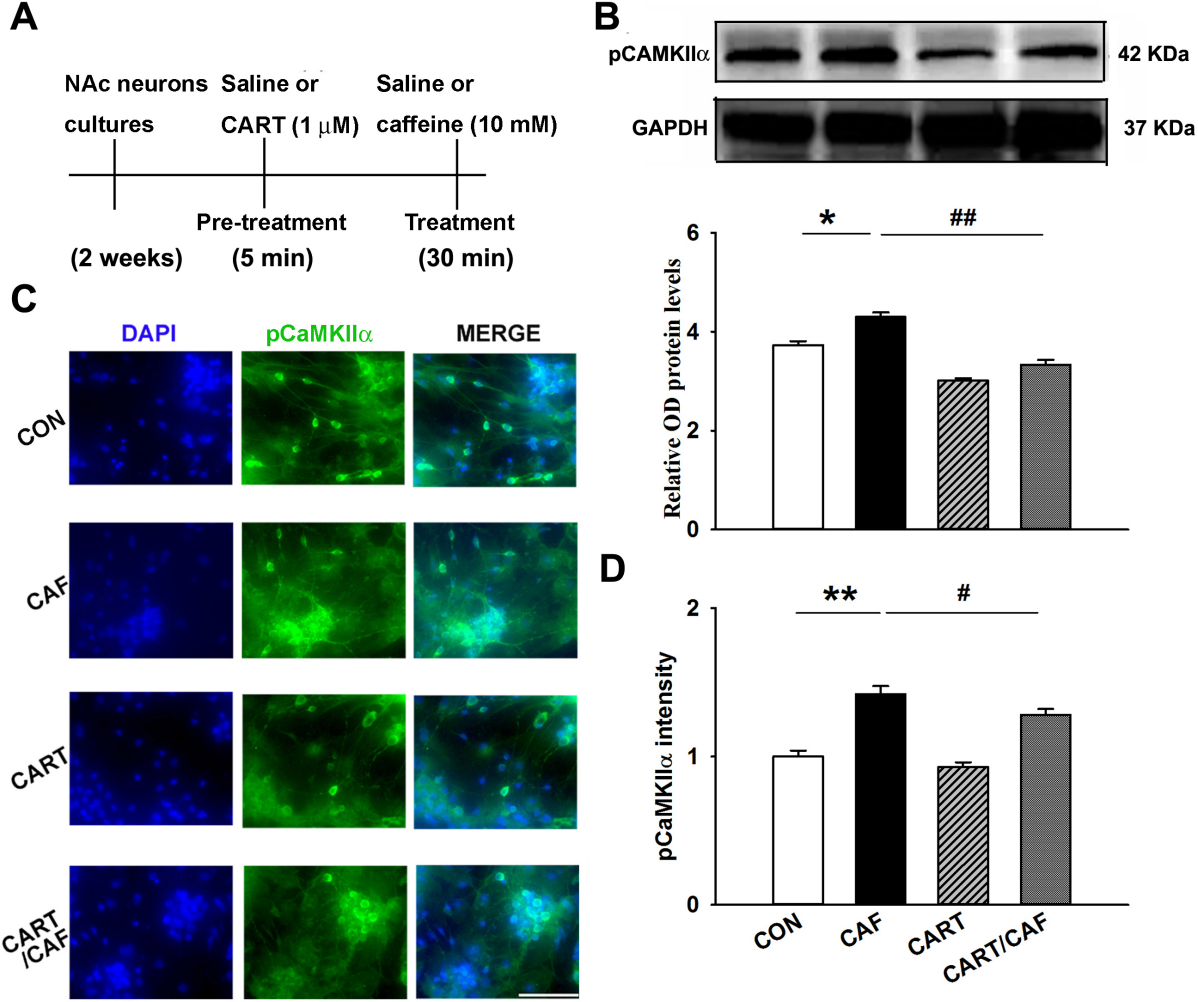
CART Peptide Decreases Caffeine-induced CaMKIIα Phosphorylation in Cultured Accumbal Neurons. (**a**) Experimental timeline. (**b**) A representative western blot labeled with antibodies against pCaMKIIα. The membranes were scanned, and the band intensities were quantified by measuring the relative density (relative OD) of the immunoreactive signals of pCaMKIIα to GAPDH. (**c**) Representative immunofluorescence images of accumbal neurons stained with antibodies against pCaMKIIα. (**d**) Bar graph showing quantification of pCaMKIIα expression in cultured NAc cells (c). The data are presented as the group means ± SEM (n = 4/group). *p<0.05 and **p<0.01 compared to the saline group; ^#^*p*<0.05 and ^##^*p*<0.01 compared to the caffeine group. Symbols indicate significant differences as revealed by two-way ANOVA followed by Bonferroni’s *post hoc* test. Symbols represent different groups: □, saline (intra-NAc)-saline (p.o.); ■, saline-caffeine; ▩, CART 55–102 (0.08 μM/side)-saline; ▨, CART 55-102-caffeine.

The western blot and immunofluorescence analyses showed significantly increased phosphorylation of CaMKIIα in NAc neurons after treatment with caffeine compared with the control (saline) treatment (*n* = 4, *p*<0.05 and *p*<0.01, Fig 4B, 4C and 4D); however, CART 55–102 peptide treatment significantly blocked the caffeine-induced increase in CaMKIIα phosphorylation in cultured NAc neurons compared with caffeine treatment (*n* = 4, *p*<0.01 and *p*<0.05, Fig 4B, 4C and 4D).

## Discussion

These studies revealed that CART peptides inhibit CREB phosphorylation in the rat NAc shell by decreasing intracellular Ca^2+^ concentration fluctuations, CaMKIIα phosphorylation, the pCaMKIIα-D3R interaction and cAMP-PKA signaling, all of which would otherwise occur in response to caffeine administration. Further intra-accumbal shell injections of CART 55–102 peptide decreased locomotor behavior following oral administration of caffeine. It was previously found that CREB activity in the NAc shell is important for the activity of psychostimulants [35–37]. After caffeine administration, CART peptides are more highly expressed in the NAc shell than in the NAc core [15,29]. The NAc shell is reported to have a major role in the integration of brain reward mechanisms and the functions of core structures that are engaged in motor-associated outputs [38]. CART mRNA is more significantly colocalized with both D_3_R transcripts in the NAc shell than in the NAc core [23,39]. Clozapine reduces the levels of CART mRNA in the NAc shell by approximately 40% but does not alter the CART mRNA levels in the NAc core [39]. Our data provide further evidence of the effects of CART peptides in the NAc shell on the action of psychostimulants.

In the present experiments, we found that caffeine increased the levels of cAMP, PKA activity and CREB phosphorylation in the rat NAc. However, these stimulatory effects were blocked by microinjection of the active CART peptide at this site. These results suggest the inhibitory effects of CART peptide on locomotor behavior. Based on these results, the inhibitory effects of CART peptide may be mediated by the CART peptide-induced decrease in CREB activation in the NAc. Whether this inhibitory effect of CART peptide on the caffeine-induced increase in pCREB levels is mediated by the direct interactions of CART peptide with Ca^2+^ signaling pathway members in the NAc is presently unknown. We have previously found that active CART peptide directly inhibited Ca^2+^ influx in rat NAc neurons in a dose-dependent manner. Moreover, we found that active CART peptide blocked the cocaine-induced increase in Ca^2+^ influx in these neurons [32]. Accordingly, the present data demonstrate that CART peptides attenuated the caffeine-mediated enhancement of depolarization-induced Ca^2+^ influx in rat NAc neurons. In recent years, investigations have demonstrated that CaMKs have a primary role in the phosphorylation of CREB and in the regulation of sensitization-dependent neuronal gene expression in NAc neurons. For example, upregulating CaM levels in the NAc resulted in an enhancement of psychostimulant-induced locomotion [40], whereas downregulating CaM levels at this site produced the opposite effect [8,9,11]. Our present findings show for the first time that CREB activation by caffeine in the rat NAc is inhibited by direct microinjection of CART peptides at this site. These findings further suggest that the suppressive effect of CART peptides in the NAc on caffeine-induced behavioral sensitization may be mediated by the interruption of Ca^2+^ signaling and cAMP/PKA signaling, thereby resulting in the inhibition of CREB phosphorylation at this site. Whether the detailed molecular mechanism by which CART regulates Ca^2+^/cAMP/PKA/pCREB signaling is associated with dopamine receptors is unknown.

Our previous data showed that active CART peptide directly inhibited the cocaine-induced enhancement of D_1_R, D_2_R, and D_3_R phosphorylation as well as cAMP/PKA and ERK signaling. Moreover, we demonstrated that D_3_R is involved in the inhibitory regulation of CART in a dose-dependent manner through the interactions of CaMKIIα with D_3_R in rat NAc neurons [31]. D_3_R activity inhibits adenylyl cyclase and CREB phosphorylation in heterologous expression systems [41] and promotes GABA release [42,43]. CART was found to be highly concentrated in medium spiny projection neurons that contain GABA, which inhibits the effects of dopamine via the activation of κ-receptors [44]. In this study, we showed that the decreases in the intracellular Ca^2+^ concentration and CaMKIIα phosphorylation interfered with the recruitment of pCaMKIIα to D_3_R. The decrease in pCaMKIIα–D_3_R interactions led to the dephosphorylation of D_3_R and a reduction in the phosphorylation of CREB. Thus, the detailed molecular mechanisms by which CART inhibits locomotor activity and pCREB signaling are associated with the activation of D_3_Rs in the rat NAc. Further research is needed to better understand the molecular processes involving CART and D_3_R-GABA signaling.

The present findings are the first to show that repeated microinjection of this peptide into the NAc further inhibits locomotor behavior that would otherwise be induced by repeated oral administration of caffeine. Injection of CART peptides into either the VTA or the NAc attenuates the locomotor activity produced by systemic cocaine and amphetamine administration [25,26]. Coincidentally, injection of CART peptides into the ventral pallidum inhibited cocaine-induced locomotion [45]. However, repeated injection of CART peptides into the VTA produced an increase in locomotor activity [46]. The locomotor effects of psychostimulants were reduced in CART knockout mice [47]. We cannot completely exclude the possibility that CART may have a positive modulatory role in the locomotor and motivational properties of psychostimulants. Taken together, our present results indicate that CART peptides in the NAc may suppress repeated caffeine administration-induced behavioral sensitization. At a minimum, the CART-associated neuronal circuitry that mediates the reinforcing properties of psychostimulants is intact.

In conclusion, these results indicate that microinjection of CART peptide into the NAc inhibits locomotor behavior in response to caffeine by suppressing the caffeine-induced activation of Ca^2+^ signaling, pCaMKIIα-D_3_R interactions and D_3_R-cAMP/PKA/pCREB signaling. These findings are consistent with the proposal that CART peptides in the NAc shell are important negative modulators involved in the effects of caffeine on locomotor activity and behavioral sensitization. Accordingly, CART peptides may ultimately prove to be potential targets for the design of pharmacotherapies to treat addiction.

## Supporting Information

S1 Data Raw data. (RAR)

S1 Fig The protocol for the care and use of animals in this study. (TIF)

S2 Fig Locomotor activity and behavioral sensitization were significantly induced by caffeine (30 mg/kg). Data are presented as means ± SEM. ^###^p<0.001 compared with the saline group. *p<0.05 compared day 1 of caffeine treatment.
(TIF)
