# Supplementary figures and images for: Decreased Caffeine-Induced Locomotor Activity via Microinjection of CART Peptide into the Nucleus Accumbens Is Linked to Inhibition of the pCaMKIIa-D3R Interaction
Source: PLoS One. 2016 Jul 12;11(7):e0159104. doi: 10.1371/journal.pone.0159104 (PMC4942143; doi:10.1371/journal.pone.0159104)

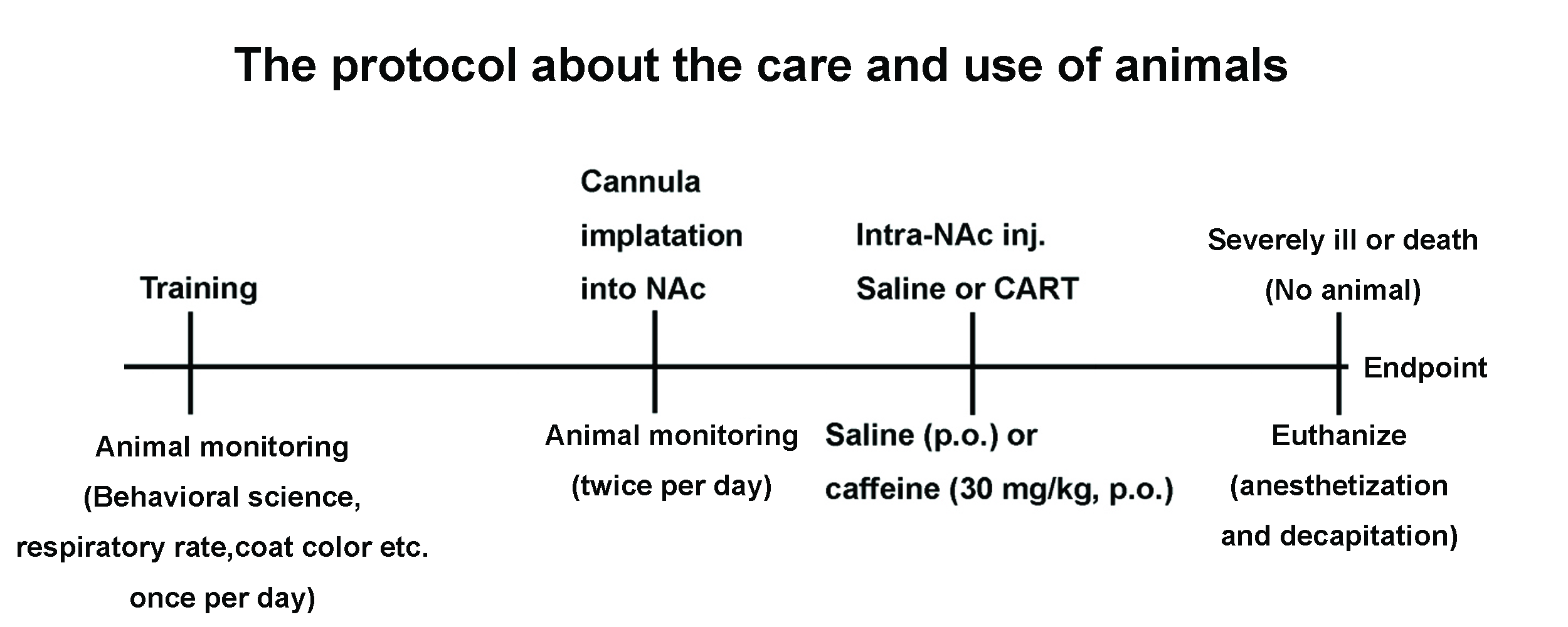

Supplement: S1 Fig — (TIF) [file pone.0159104.s002.tif]

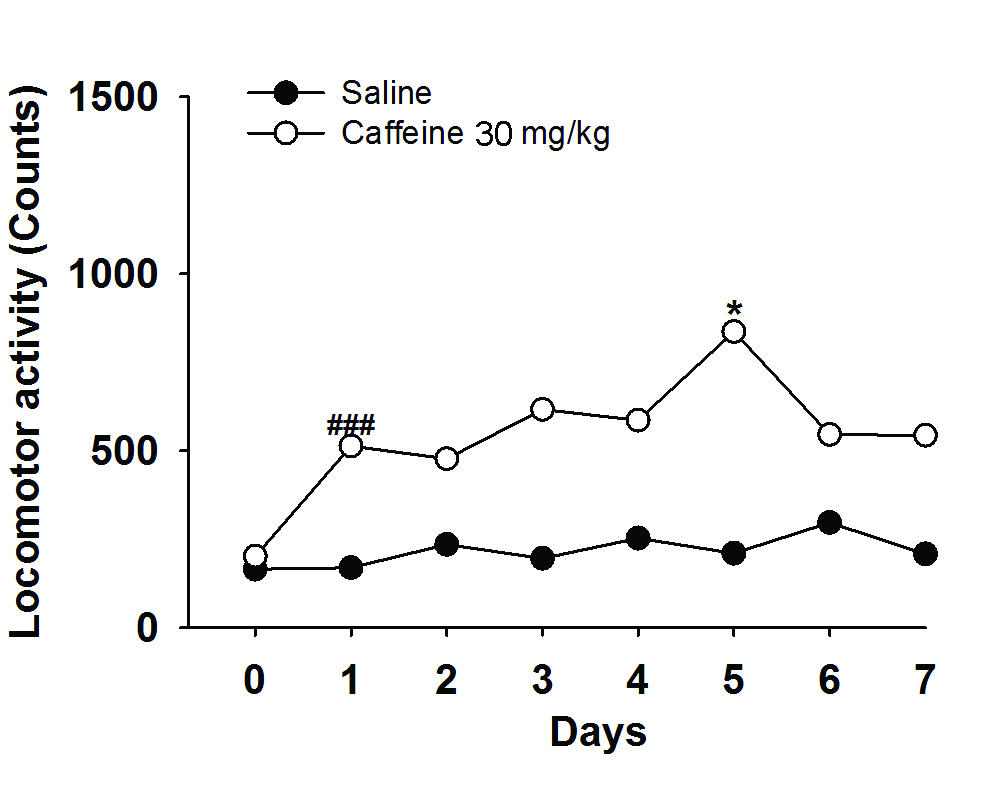

Supplement: S2 Fig — Data are presented as means ± SEM. ###p<0.001 compared with the saline group. *p<0.05 compared day 1 of caffeine treatment. (TIF) [file pone.0159104.s003.tif]
